# Stage-specific high-temperature stress reduces grain yield by disrupting photosynthesis and antioxidant defenses in summer maize

**DOI:** 10.3389/fpls.2026.1899671

**Published:** 2026-07-24

**Authors:** Ruilian Zhang, Xiaomin Shang, Kailin Dong, Jing Li, Yang Liu, Mengyuan Cao, Abdul Rehman, Wenyang Li

**Affiliations:** 1 College of Agronomy, Anhui Science and Technology University, Chuzhou, China; 2 Maize Germplasm Resources Center of Anhui Province, Chuzhou, China

**Keywords:** high-temperature stress, leaf senescence, maize, photosynthetic characteristics, yield

## Abstract

High-temperature stress has become increasingly frequent and intense in the Huanghuaihai summer maize region, posing a serious threat to maize production. In this study, two maize cultivars, Anke985 and Helian1589, were subjected to high-temperature stress at four key growth stages: large whorl (V12), tasseling (VT), blister (R2), and milk (R3), with ambient conditions as the control (CK). High-temperature stress was imposed at different growth stages, and key physiological and biochemical traits, including photosynthetic characteristics, chlorophyll fluorescence parameters, antioxidant enzyme activities, dry matter accumulation, and yield components were investigated. High-temperature stress significantly reduced grain yield by decreasing kernel number per ear, kernel number per row, and 100-kernel weight. Correspondingly, grain yield under V12-HT, VT-HT, R2-HT, and R3-HT treatments decreased by 40.03%, 45.13%, 21.72%, and 16.82%, respectively, compared with the control. High-temperature stress impaired photosynthetic performance and antioxidant defense, accompanied by reduced chlorophyll fluorescence efficiency and enhanced oxidative damage. Dry matter allocation to grains was also reduced, particularly under V12-HT and VT-HT, with reductions of 26.48% and 27.68%, respectively, indicating restricted assimilate transport to developing grains. Both maize cultivars showed consistent response patterns. Overall, the adverse effects were most pronounced when high-temperature stress occurred at the V12 and VT stages. Collectively, stage-specific high-temperature stress impaired photosynthesis and antioxidant defense systems, leading to accelerated leaf senescence, reduced dry matter accumulation, and restricted assimilate allocation to developing kernels, thereby ultimately decreasing grain yield.

## 1 Introduction

Maize is one of the most important cereal crops in China and plays a critical role in ensuring national food security (Rezaei et al., 2023). In recent decades, global climate warming has intensified climate variability and increased the frequency of extreme weather events, posing serious threats to agricultural production worldwide. Although maize is a warm-season crop, it remains highly sensitive to high-temperature stress across its developmental stages, and a daytime temperature above 35 °C is widely recognized as the critical threshold that induces heat injury (Li et al., 2023). High-temperature stress has therefore become one of the major abiotic constraints limiting crop productivity.

Previous studies have demonstrated that high-temperature stress reduces leaf area development, impairs photosynthetic capacity and key metabolic enzyme activities, thereby restricting dry matter accumulation and ultimately reducing grain yield in maize (Waqas et al., 2021; Li et al., 2022). In addition, high-temperature stress restricts assimilate transport from source leaves to developing kernels, resulting in reduced kernel set and lower grain yield. Photosynthesis is the primary physiological process determining biomass accumulation and yield formation in maize. High-temperature stress impairs photosynthetic performance by altering leaf gas exchange parameters and reducing chlorophyll fluorescence efficiency, while accelerating chlorophyll degradation and premature leaf senescence (Yu et al., 2017a). This dual negative effect ultimately limits carbon assimilation and grain production. Beyond inhibiting photosynthesis, high-temperature stress disrupts cellular redox homeostasis by inducing excessive accumulation of reactive oxygen species (ROS). Although antioxidant enzymes, including superoxide dismutase (SOD), peroxidase (POD), and catalase (CAT), normally function to scavenge excess ROS, prolonged high-temperature stress weakens the antioxidant defense system, aggravates membrane lipid peroxidation, accelerates leaf senescence, and further impairs photosynthetic performance (Hasanuzzaman et al., 2020). Previous studies have generally identified the tasseling stage as the most heat-sensitive period in maize because high-temperature stress during this stage impairs leaf photosynthesis, reduces pollen viability, prolongs the anthesis–silking interval, and decreases kernel set, ultimately resulting in yield loss (Mu et al., 2022, 2023; Yu et al., 2017b; Song et al., 2014). Consequently, most previous studies have primarily focused on high-temperature stress during the tasseling stage, whereas the physiological responses to high-temperature stress imposed before and after tasseling remain less well understood.

As global warming continues to intensify, high-temperature stress is no longer confined to the tasseling stage but exerts adverse impacts on maize at multiple developmental stages. Recent studies have shown that high-temperature stress frequently occurs during multiple developmental stages, including the jointing-to-tasseling stage (Yao et al., 2019), blister stage (Yang et al., 2017), and milk stage (Djanaguiraman et al., 2020). In rice, wheat, and other field crops, plant responses to high-temperature stress have been shown to exhibit strong growth-stage specificity, with crop sensitivity varying considerably among developmental stages (Lawas et al., 2018; Bergkamp et al., 2018; Talukder et al., 2014). Nevertheless, studies investigating the stage-specific effects of high-temperature stress across multiple growth stages in summer maize remain limited, particularly regarding the coordinated relationships among photosynthetic performance, antioxidant defense systems, leaf senescence, assimilate allocation, and grain yield formation (Edreira et al., 2011; Bheemanahalli et al., 2022).

Physiological metabolism and source–sink relationships differ substantially among maize developmental stages, suggesting that the responses of photosynthesis, antioxidant defense and assimilate allocation to high-temperature stress may also vary with developmental stage (Fernie et al., 2020). However, whether differences in developmental stage alter the coordination among photosynthetic performance, antioxidant defense, leaf senescence and assimilate allocation under high-temperature stress remains unclear. Clarifying these stage-dependent physiological responses is essential for understanding the mechanisms responsible for differential yield loss. Therefore, this study compared the effects of high-temperature stress imposed at four representative developmental stages: the large whorl stage, tasseling stage, blister stage, and milk stage, on photosynthetic performance, antioxidant defense, leaf senescence, assimilate allocation, and grain yield. The objective was to clarify the physiological mechanisms underlying stage-specific heat injury and to provide a theoretical basis for improving the heat resistance and yield stability of summer maize under future warming scenarios.

## 2 Materials and methods

### 2.1 Experimental design

In this study, a field experiment was conducted in 2024–2025 at the experimental farm of Anhui Science and Technology University (117.56°E, 32.88°N). Two maize cultivars, AK985 and HL1589, were used as experimental materials. High-temperature shelters were used to simulate high-temperature stress. Four high-temperature treatments were applied at the large whorl stage (V12-HT), the tasseling stage (VT-HT), the blister stage (R2-HT), and the milk stage (R3-HT), with normal temperature treatment as the control (CK). The effects of high-temperature stress on photosynthetic characteristics, leaf senescence, and grain yield of summer maize were studied. This study provides a theoretical basis for stress-resistant cultivation of maize.

**Figure 1:**
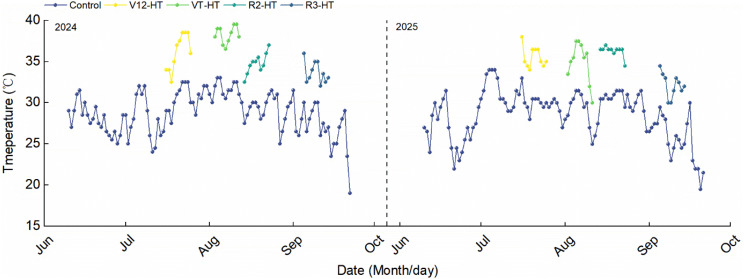
Daily mean temperature under different treatments during the high-temperature treatment period. The blue line represents the ambient control (CK), whereas the yellow, green, cyan, and dark blue lines represent the V12-HT, VT-HT, R2-HT, and R3-HT treatments, respectively. Temperature was continuously monitored throughout the experimental period in 2024 and 2025. Each high-temperature treatment lasted for 10 consecutive days.

The high-temperature treatment was conducted according to the methods of Yu et al. (2023). A steel-frame high-temperature shelter (3 m × 2.5 m × 3 m) was built. The shelter was covered with plastic film with a light transmittance above 95%. The plastic film on all sides could be lifted for air exchange. The high-temperature treatment lasted for 10 days. The treatment was applied for 10 h per day (8:00–18:00). At other times, plants grew under natural temperature. After the treatment, the shelters were removed. Plants then grew under natural conditions until maturity. The experiment was arranged in a randomized complete block design with three replicates. Each plot consisted of five rows (5 m in length with a row spacing of 0.6 m) at a planting density of 63,000 plants ha^-^¹. For physiological measurements, five representative plants were randomly selected from the central rows of each plot, and the mean value of these five plants was used as one biological replicate for statistical analysis. The soil organic matter content was 16.65 mg kg^-^¹. The alkali-hydrolyzable nitrogen content was 72.75 mg kg^-^¹. The available potassium content was 96.05 mg kg^-^¹. The available phosphorus content was 17.45 mg kg^-^¹. Other management practices followed conventional cultivation methods. During the treatment period, air temperature and relative humidity were continuously monitored using a temperature and humidity recorder. Across the high-temperature treatment periods in the 2024 and 2025 growing seasons, the mean air temperature inside the high-temperature shelter was 35 °C, which was approximately 5 ± 2 °C higher than that under the ambient control (**Figure 1**).No significant differences in relative humidity or light intensity were observed between the high-temperature treatment and the control. The VT-stage treatment is presented as a representative example (**Table 1**).

**Table 1:** Microclimatic conditions under control and high-temperature treatments during the VT stage.

Year	Treatment	Average light intensity (μmol·m^-2^·s^-1^)	Average CO_2_ concentration (μmol·mol^-1^)	Relative humidity (%)
2024	CK	1129.4a	442.8a	70.71a
	HT	1079.1b	449.1a	71.92a
2025	CK	1138.55a	446.34a	74.25a
	HT	1099.7b	452.16a	76.65a

Different lowercase letters within the same column in the same year indicate significant differences among treatments according to the LSD test (P < 0.05). CK, normal temperature; HT, high temperature.

### 2.2 Measurement and methods

#### 2.2.1 SPAD value

During the high-temperature treatment, SPAD values were measured every 5 days between 09:00 and 11:00 h. For each treatment, 15 maize plants with uniform growth were randomly selected. Every five plants were regarded as one biological replicate, resulting in three biological replicates (n = 3). Leaf SPAD values were measured using a SPAD-502 portable chlorophyll meter. At the V12 stage, measurements were performed on the 12th fully expanded leaf, whereas at the VT stage and thereafter, the ear leaf was used for SPAD measurements.

#### 2.2.2 Leaf photosynthetic parameters

During the high-temperature treatment at the VT stage, leaf gas exchange parameters were measured every 5 days between 09:00 and 11:00 h. For each treatment, 15 maize plants with uniform growth were randomly selected, and the ear leaf was used for measurements while avoiding the main leaf vein. Every five plants were regarded as one biological replicate, resulting in three biological replicates (n = 3). Net photosynthetic rate (Pn), transpiration rate (Tr), stomatal conductance (Gs), and intercellular CO_2_ concentration (Ci) were determined using a portable photosynthesis system (LI-6400). During the measurements, the photosynthetically active radiation (PAR) in the leaf chamber was maintained at 1200 μmol m^-^² s^-^¹, the reference CO_2_ concentration was set at 390 μmol mol^-^¹, the block temperature was maintained at 23.5 °C, and the air flow rate was set at 500 μmol s^-^¹.

#### 2.2.3 Chlorophyll fluorescence parameters

During the high-temperature treatment, chlorophyll fluorescence parameters were measured every 5 days. For each treatment, 15 maize plants with uniform growth were randomly selected. Every five plants were regarded as one biological replicate, resulting in three biological replicates (n = 3). At the V12 stage, measurements were performed on the 12th fully expanded leaf, whereas at the VT stage and thereafter, the ear leaf was used for measurements, avoiding the main leaf vein. Chlorophyll fluorescence parameters were measured using a MINI-PAM-II chlorophyll fluorometer. Leaves were dark-adapted for 15–20 min prior to measurement. The measuring light intensity was set to level 3, and a saturating pulse with an intensity of level 4 and a pulse duration of 0.6 s was applied. Actual photochemical efficiency (ΦPSII), maximum photochemical efficiency (Fv/Fm), photochemical quenching coefficient (qP), and non-photochemical quenching coefficient (qN) were recorded.

#### 2.2.4 Antioxidant enzyme activity and MDA content

At the R3 stage, sampling was conducted on the day following the completion of the high-temperature treatment. For each treatment, 15 maize plants with uniform growth were randomly selected. The ear leaves were collected, with every five plants pooled as one biological replicate, resulting in three biological replicates (n = 3). The collected samples were immediately frozen in liquid nitrogen and stored at −80 °C for the determination of antioxidant enzyme activities and malondialdehyde (MDA) content. MDA content was determined according to the method of Zou (2000). Superoxide dismutase (SOD) activity was determined using the nitro blue tetrazolium (NBT) photoreduction method, with one unit of enzyme activity defined as the amount of enzyme causing 50% inhibition of NBT photoreduction. Peroxidase (POD) activity was determined using the guaiacol method (Li, 2000), and catalase (CAT) activity was determined using the potassium permanganate titration method.

#### 2.2.5 Dry matter accumulation and distribution

At the VT and R6 stages, 15 maize plants with uniform growth were randomly selected from each treatment. Every five plants were regarded as one biological replicate, resulting in three biological replicates (n = 3). The aboveground parts of the plants were harvested and separated into stems, leaves, husks, cobs, and kernels. The samples were initially heated at 105 °C for 30 min and then dried at 80 °C to constant weight. The dry weight of each organ was determined, and the dry matter accumulation and distribution per plant were subsequently calculated.

#### 2.2.6 Yield and its components

At maturity, three replicate plots were sampled for each treatment. Twenty representative ears were randomly selected from each replicate plot to determine the kernel number per ear and 100-kernel weight. Grain yield per plant was calculated.

### 2.3 Method data processing and analysis

Excel 2016 and DPS 7.05 were used for data processing. Two-way analysis of variance was performed on the data. The LSD method was used for significance test. Origin 2021 was used for plotting. The data of the measured parameters presented in this study represent the mean values of two growing seasons (2024–2025) and two maize cultivars to eliminate the random effects of year and cultivar.

## 3 Results

### 3.1 Yield and its components

**Figure 2:**
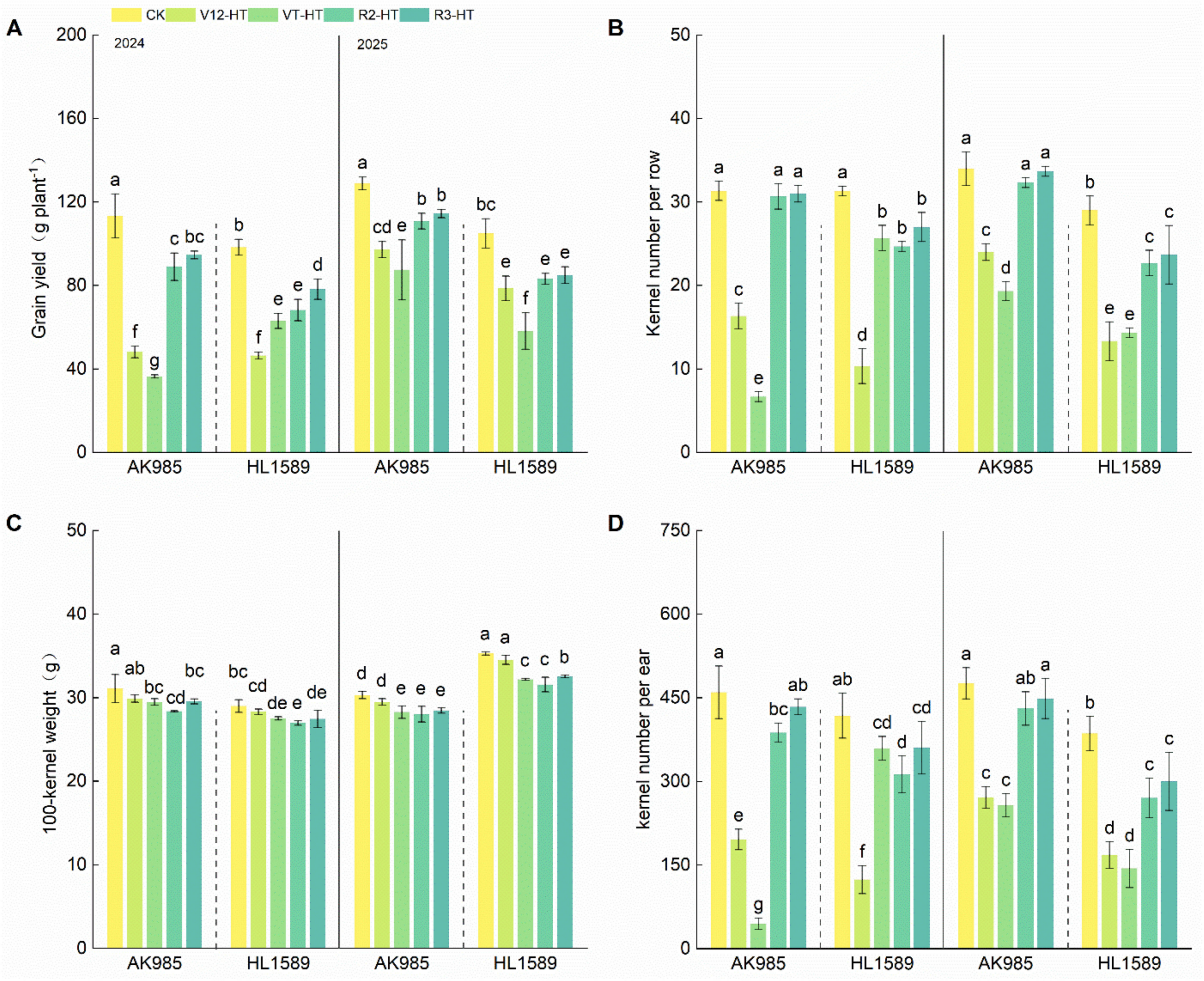
Effects of stage-specific high-temperature stress on grain yield and yield components. **(A)** Grain yield; **(B)** Kernel number per row; **(C)** 100-kernel weight; **(D)** Kernel number per ear. Data are presented as the mean ± SD (n = 3). Different lowercase letters indicate significant differences among treatments within the same cultivar and year according to the LSD test (*P* < 0.05). CK, normal temperature; V12-HT, high-temperature stress imposed at the V12 stage; VT-HT, high-temperature stress imposed at the VT stage; R2-HT, high-temperature stress imposed at the R2 stage; R3-HT, high-temperature stress imposed at the R3 stage.

High-temperature stress at different stages significantly reduced kernel number per row, kernel number per ear, and 100-kernel weight in both maize cultivars, ultimately leading to yield reduction (**Figure 2**). Compared with the control, grain yield under V12-HT, VT-HT, R2-HT, and R3-HT decreased by 40.03%, 45.13%, 21.72%, and 16.82%, respectively. Kernel number per row decreased by 49.59%, 47.63%, 12.53%, and 8.56%, while kernel number per ear decreased by 56.28%, 52.54%, 20.10%, and 11.85% under the four treatments. The corresponding reductions in 100-kernel weight were 2.80%, 6.46%, 8.50%, and 6.04%. Overall, high-temperature stress significantly affected maize yield components, with kernel number per ear showing the greatest reduction, followed by 100-kernel weight. These results indicate that high-temperature stress reduced grain yield primarily by decreasing kernel number per ear and kernel weight, with the most pronounced reductions occurring when stress was imposed at the V12 and VT stages.

### 3.2 Leaf SPAD value of maize

**Figure 3:**
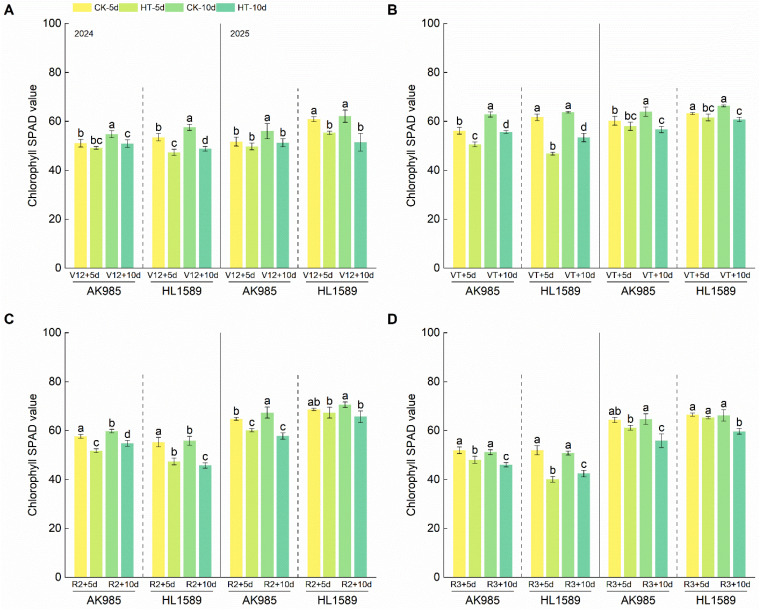
Effects of stage-specific high-temperature stress on leaf SPAD values. **(A)** V12 stage; **(B)** VT stage; **(C)** R2 stage; **(D)** R3 stage. Data are presented as the mean ± SD (n = 3). Different lowercase letters indicate significant differences among treatments within the same cultivar, year, and sampling time according to the LSD test (*P* < 0.05). CK: normal temperature; HT, high-temperature stress.

High-temperature stress imposed at different growth stages significantly affected the leaf SPAD value of maize, with consistent trends observed across the two experimental years (**Figure 3**). Compared with the control, SPAD values decreased by 5.93%, 7.52%, 6.96%, and 5.52% after 5 days of high-temperature treatment at the V12, VT, R2, and R3 stages, respectively, and by 12.83%, 17.12%, 12.66%, and 14.39% after 10 days. High-temperature stress at all four growth stages significantly reduced leaf SPAD values, and the magnitude of the reduction increased with the duration of stress. Among the four growth stages, the VT stage was the most sensitive to high-temperature stress, exhibiting the greatest decline in leaf SPAD values.

### 3.3 Photosynthetic characteristics of ear leaf in maize

#### 3.3.1 Net photosynthetic rate

**Figure 4:**
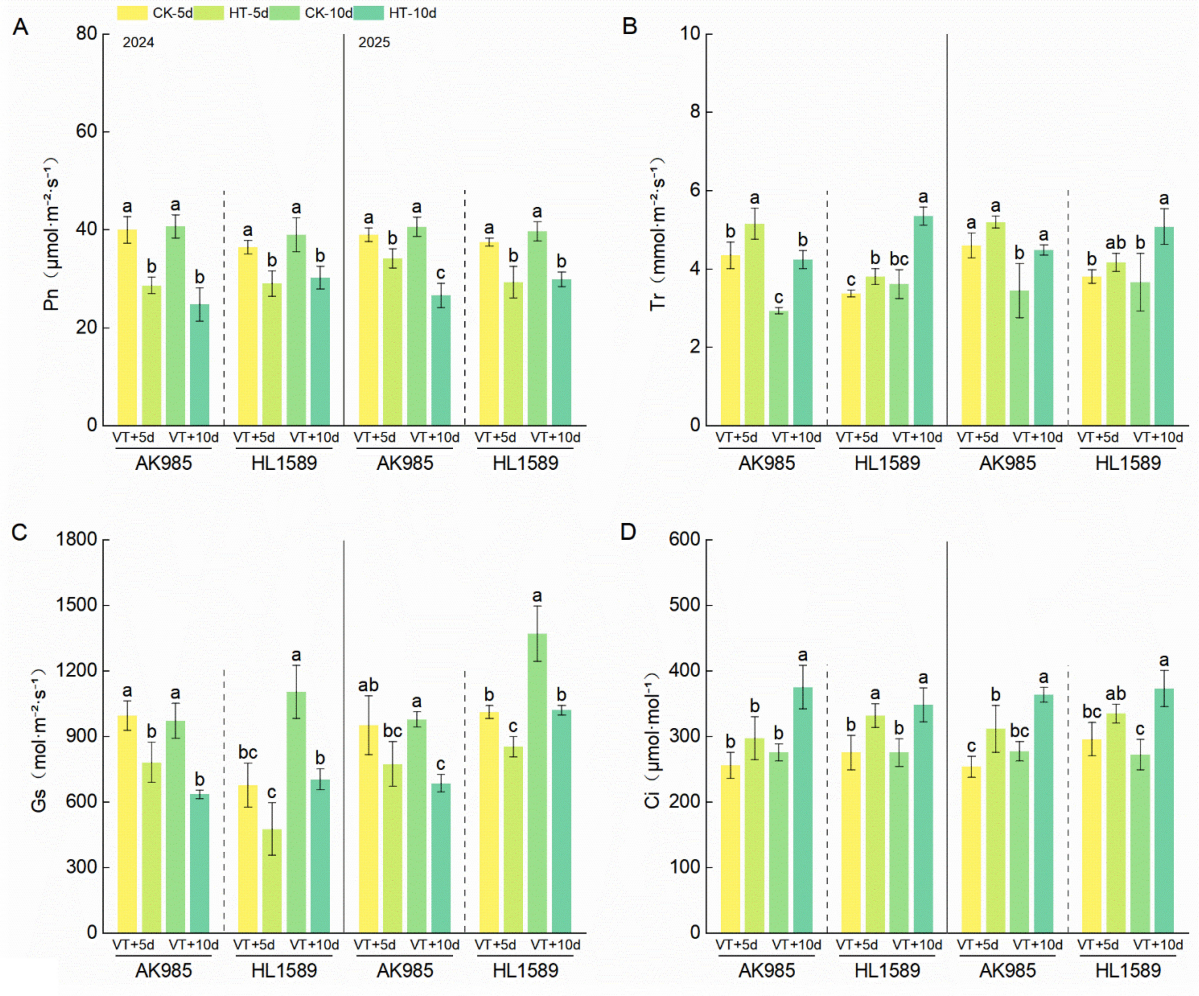
Effects of high-temperature stress at the VT stage on photosynthetic characteristics of ear leaves. **(A)** Net photosynthetic rate (Pn); **(B)** Transpiration rate (Tr); **(C)** Stomatal conductance (Gs); **(D)** Intercellular CO_2_ concentration (Ci). Data are presented as the mean ± SD (n = 3). Different lowercase letters indicate significant differences among treatments within the same cultivar, year, and sampling time according to the LSD test (*P* < 0.05).

Compared with the control, high-temperature stress significantly reduced Pn in both maize cultivars, with similar response patterns observed across the two experimental years (**Figure 4A**). At the VT stage, Pn decreased by 20.69% and 30.19% after 5 and 10 days of high-temperature stress, respectively, and the reduction became more pronounced with stress duration.

#### 3.3.2 Transpiration rate

Compared with the control, high-temperature stress significantly increased Tr in both maize cultivars, with similar response patterns observed across the two experimental years (**Figure 4B**). At the VT stage, Tr increased by 13.40% and 40.37% after 5 and 10 days of high-temperature stress, respectively, showing a stronger increase with prolonged stress duration.

#### 3.3.3 Stomatal conductance

Compared with the control, high-temperature stress significantly reduced Gs in both maize cultivars, with similar response patterns observed across the two experimental years (**Figure 4C**). At the VT stage, Gs decreased by 21.35% and 31.57% after 5 and 10 days of high-temperature stress, respectively, with a greater reduction under prolonged stress.

#### 3.3.4 Intercellular CO_2_ concentration

Compared with the control, high-temperature stress significantly increased Ci in both maize cultivars, with similar response patterns observed across the two experimental years (**Figure 4D**). At the VT stage, Ci increased by 18.20% and 32.57% after 5 and 10 days of high-temperature stress, respectively, and showed a stronger response under prolonged stress.

### 3.4 Chlorophyll fluorescence parameters of maize leaves

#### 3.4.1 Actual photochemical efficiency

**Figure 5:**
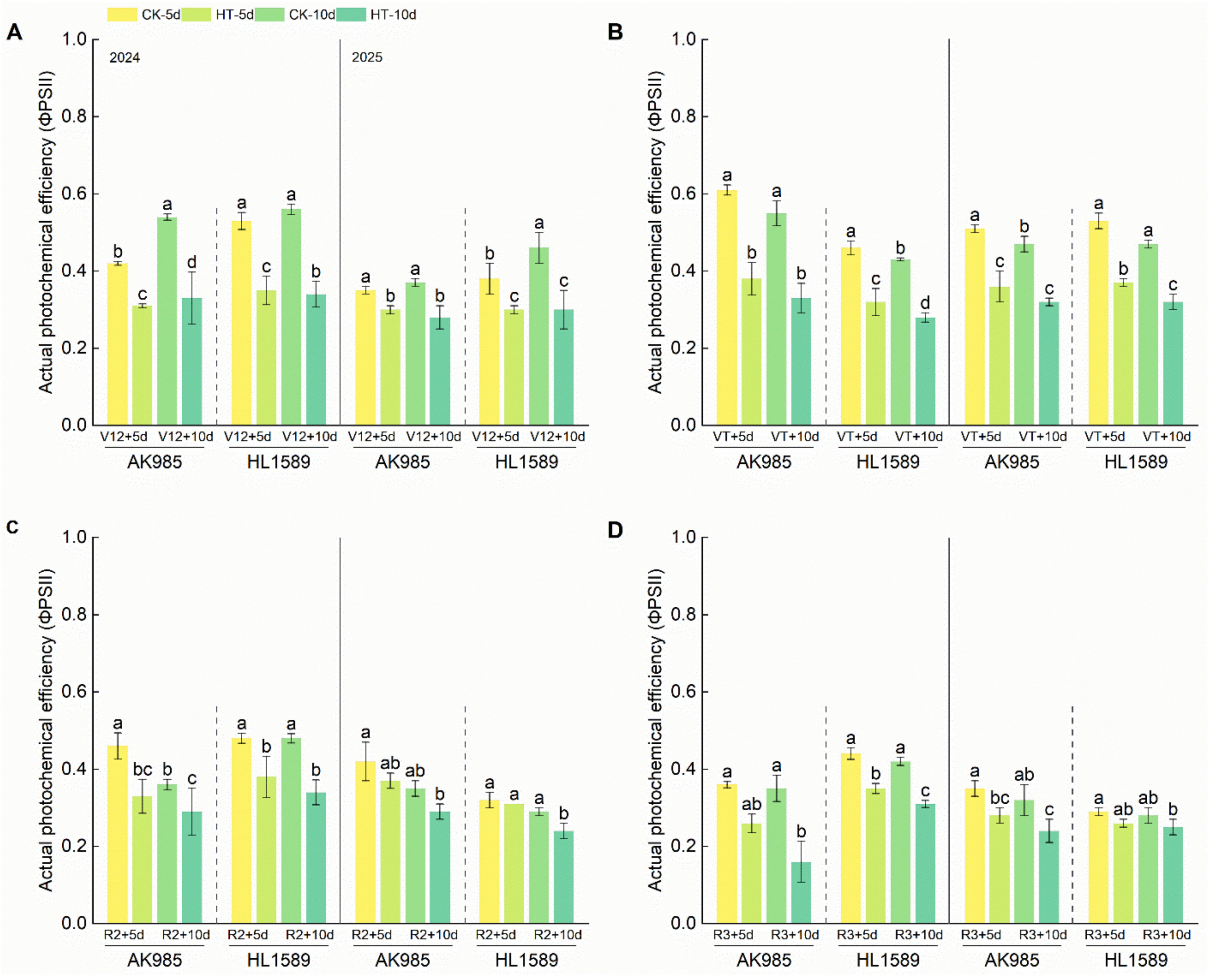
Effects of stage-specific high-temperature stress on the actual photochemical efficiency of PSII. **(A)** V12 stage; **(B)** VT stage; **(C)** R2 stage; **(D)** R3 stage. Data are presented as the mean ± SD (n = 3). Different lowercase letters indicate significant differences among treatments within the same cultivar, year, and sampling time according to the LSD test (P < 0.05).

Compared with the control, high-temperature stress at different developmental stages significantly reduced ΦPSII in both maize cultivars, with consistent response patterns across the two experimental years (**Figure 5**). At 5 days of stress, ΦPSII decreased by 23.88%, 31.94%, 16.03%, and 19.65% under V12-HT, VT-HT, R2-HT, and R3-HT, respectively. After 10 days, the corresponding reductions were 34.33%, 34.68%, 20.75%, and 29.05%, indicating a stronger decline with prolonged stress, particularly at the V12 and VT stages.

#### 3.4.2 Maximum photochemical efficiency

**Figure 6:**
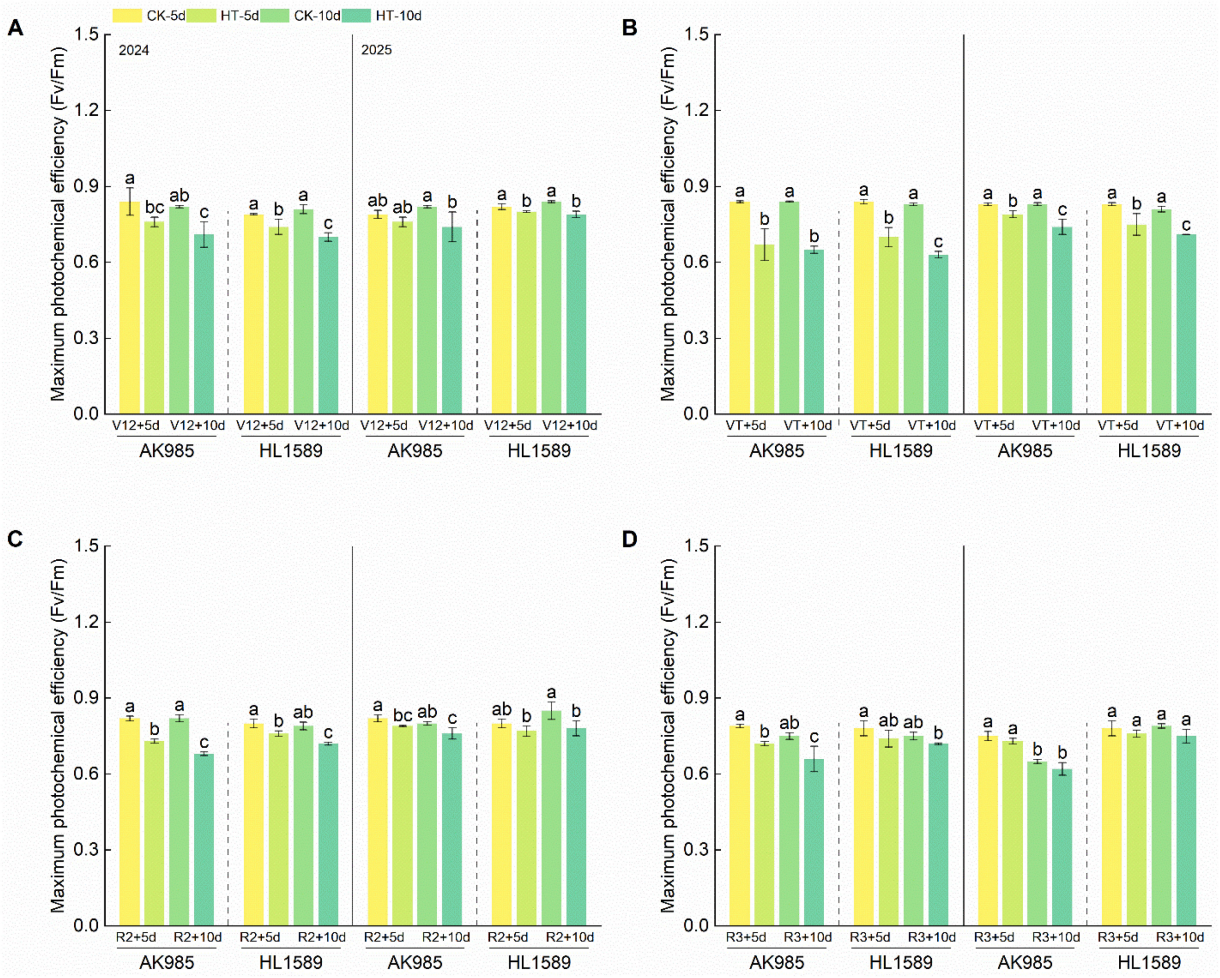
Effects of high-temperature stress on the maximum photochemical efficiency of PSII. **(A)** V12 stage; **(B)** VT stage; **(C)** R2 stage; **(D)** R3 stage. Data are presented as the mean ± SD (n = 3). Different lowercase letters indicate significant differences among treatments within the same cultivar, year, and sampling time according to the LSD test (P < 0.05).

Compared with the control, high-temperature stress at different developmental stages significantly reduced Fv/Fm in both maize cultivars, with consistent response patterns across the two experimental years (**Figure 6**). After 5 days of high-temperature stress, Fv/Fm decreased by 5.61%, 12.73%, 5.81%, and 4.86% under the V12-HT, VT-HT, R2-HT, and R3-HT treatments, respectively. After 10 days, the corresponding reductions were 10.27%, 17.35%, 9.71%, and 6.39%, with greater reductions under prolonged stress, particularly at the V12 and VT stages.

#### 3.4.3 Photochemical quenching coefficient

**Figure 7:**
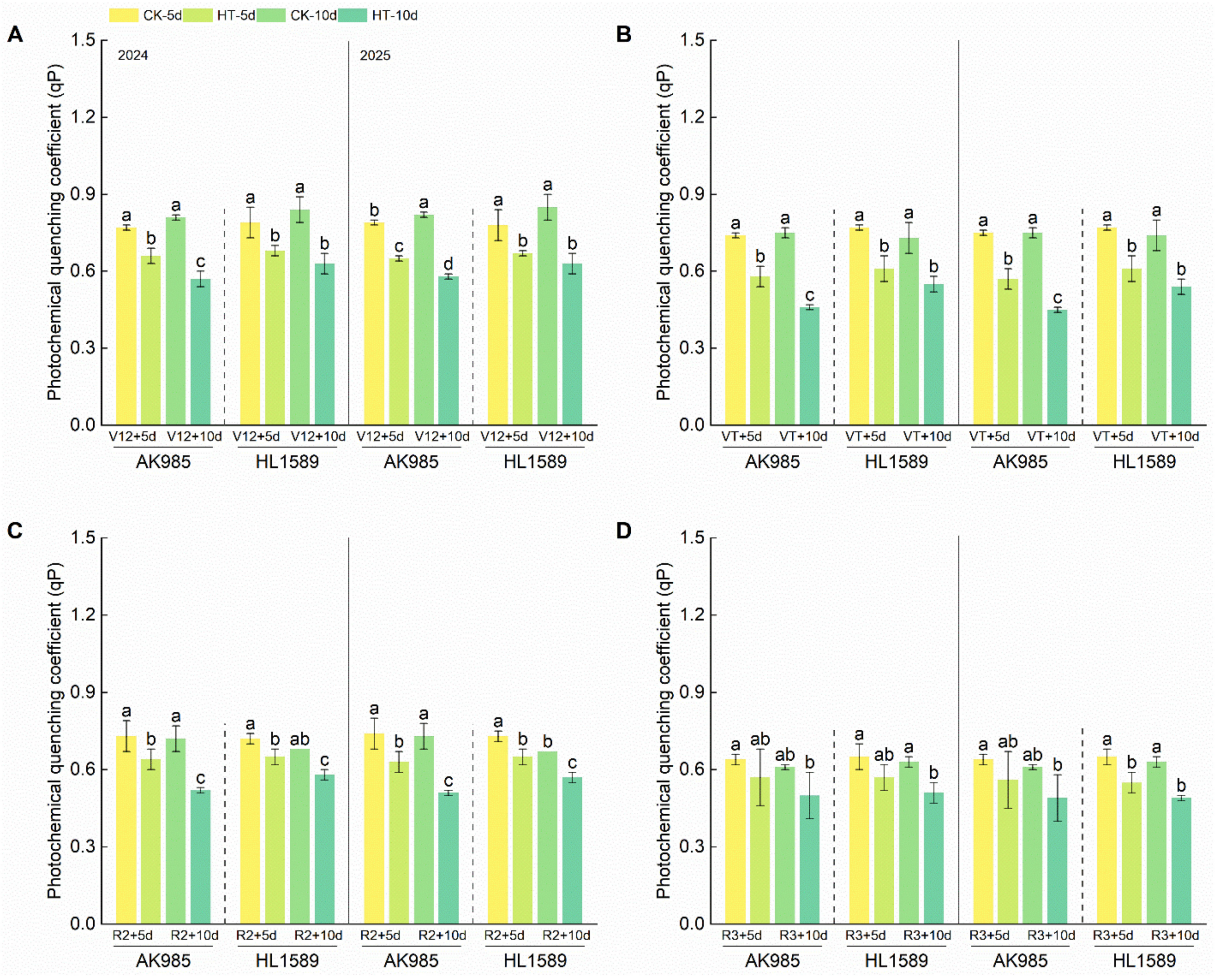
Effects of high-temperature stress on the photochemical quenching coefficient. **(A)** V12 stage; **(B)** VT stage; **(C)** R2 stage; **(D)** R3 stage. Data are presented as the mean ± SD (n = 3). Different lowercase letters indicate significant differences among treatments within the same cultivar, year, and sampling time according to the LSD test (P < 0.05).

Compared with the control, high-temperature stress at different developmental stages significantly reduced qP in both maize cultivars, with consistent response patterns across the two experimental years (**Figure 7**). After 5 days of high-temperature stress, qP decreased by 15.01%, 21.80%, 11.97%, and 12.79% under the V12-HT, VT-HT, R2-HT, and R3-HT treatments, respectively. After 10 days, the corresponding reductions were 27.45%, 32.60%, 21.89%, and 19.75%, with greater reductions under prolonged stress, particularly at the V12 and VT stages.

#### 3.4.4 Non-photochemical quenching coefficient

**Figure 8:**
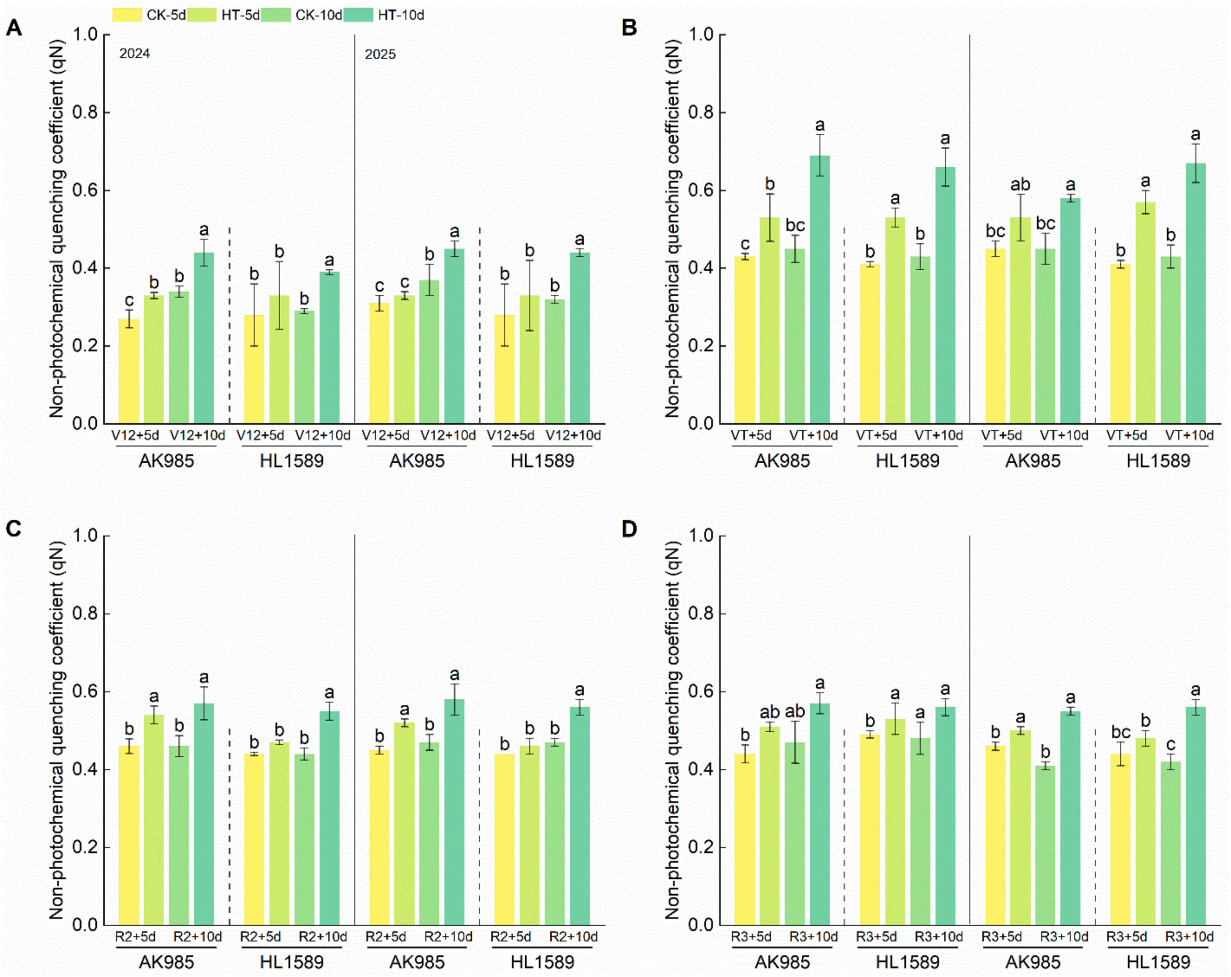
Effects of high-temperature stress on the non-photochemical quenching coefficient. **(A)** V12 stage; **(B)** VT stage; **(C)** R2 stage; **(D)** R3 stage. Data are presented as the mean ± SD (n = 3). Different lowercase letters indicate significant differences among treatments within the same cultivar, year, and sampling time according to the LSD test (P < 0.05).

Compared with the control, high-temperature stress at different developmental stages significantly increased qN in both maize cultivars, with consistent response patterns across the two experimental years (**Figure 8**). After 5 days of high-temperature stress, qN increased by 16.10%, 27.34%, 11.09%, and 10.47% under the V12-HT, VT-HT, R2-HT, and R3-HT treatments, respectively. After 10 days, the corresponding increases were 30.76%, 47.88%, 22.87%, and 26.36%, with greater increases under prolonged stress, particularly at the V12 and VT stages.

### 3.5 Antioxidant enzyme activity and MDA content of maize leaves

#### 3.5.1 Malondialdehyde

**Figure 9:**
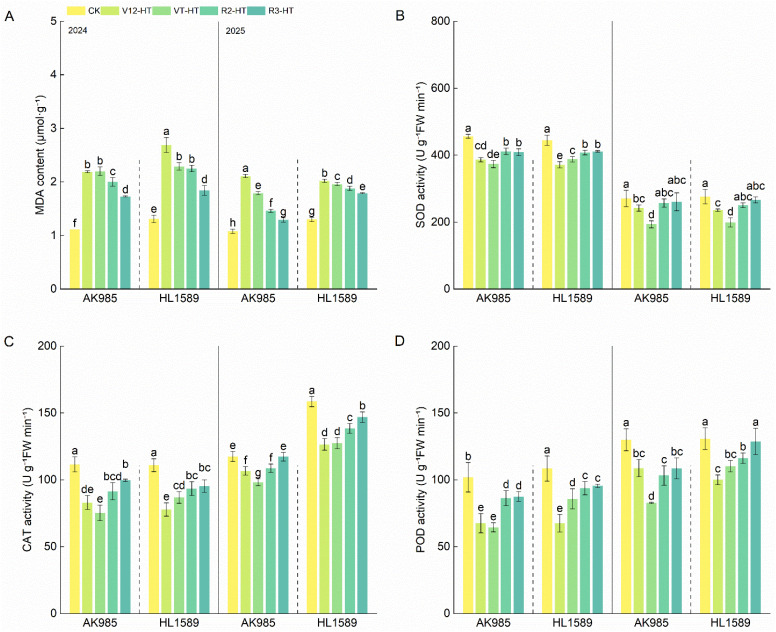
Effects of high-temperature stress on antioxidant enzyme activities and lipid peroxidation. **(A)** Malondialdehyde content; **(B)** Superoxide dismutase activity; **(C)** Catalase activity; **(D)** Peroxidase activity. Data are presented as the mean ± SD (n = 3). Different lowercase letters indicate significant differences according to the LSD test (P < 0.05).

Compared with the control, high-temperature stress at different developmental stages significantly increased MDA content in both maize cultivars, with consistent response patterns across the two experimental years (**Figure 9A**). MDA content increased by 88.35%, 72.38%, 57.94%, and 38.37% under the V12-HT, VT-HT, R2-HT, and R3-HT treatments, respectively, with greater increases at the V12 and VT stages than at the R2 and R3 stages.

#### 3.5.2 Superoxide dismutase activity

Compared with the control, high-temperature stress at different developmental stages significantly reduced SOD activity in both maize cultivars, with consistent response patterns across the two experimental years (**Figure 9B**). SOD activity decreased by 14.25%, 21.83%, 8.14%, and 6.26% under the V12-HT, VT-HT, R2-HT, and R3-HT treatments, respectively, with greater reductions at the V12 and VT stages than at the R2 and R3 stages.

#### 3.5.3 Catalase activity

Compared with the control, high-temperature stress at different developmental stages significantly reduced CAT activity in both maize cultivars, with consistent response patterns across the two experimental years (**Figure 9C**). CAT activity decreased by 21.22%, 22.58%, 13.52%, and 8.07% under the V12-HT, VT-HT, R2-HT, and R3-HT treatments, respectively, with greater reductions at the V12 and VT stages than at the R2 and R3 stages.

#### 3.5.4 Peroxidase activity

Compared with the control, high-temperature stress at different developmental stages significantly reduced POD activity in both maize cultivars, with consistent response patterns across the two experimental years (**Figure 9D**). POD activity decreased by 27.80%, 27.41%, 15.11%, and 11.03% under the V12-HT, VT-HT, R2-HT, and R3-HT treatments, respectively, with greater reductions at the V12 and VT stages than at the R2 and R3 stages.

### 3.6 Dry matter accumulation and distribution

**Figure 10:**
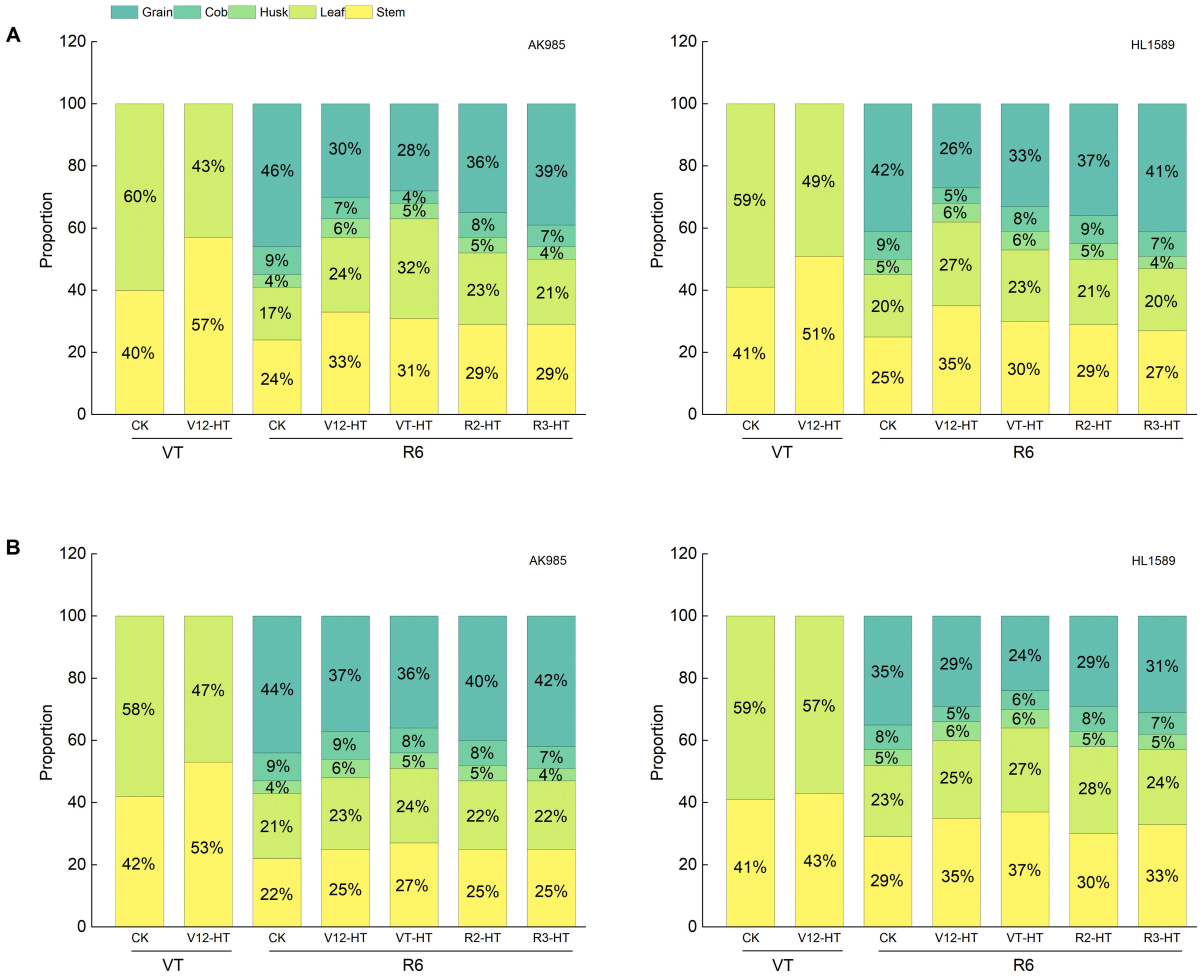
Dry matter allocation patterns under stage-specific high-temperature stress. **(A)** 2024; **(B)** 2025. Different colors represent the proportions of dry matter allocated to grain, cob, husk, leaf, and stem. Values shown within the bars indicate the percentage of total aboveground dry matter allocated to each organ.

Based on the two-year average, high-temperature stress altered dry matter allocation in maize, reducing the proportion allocated to sink organs such as cobs and kernels, while increasing the proportion allocated to vegetative organs, including stems, leaves, and husks (**Figure 10**). At the VT stage, the proportion of dry matter allocated to stems increased by 24.94% compared with the control, whereas that allocated to leaves decreased by 16.70%. At the R6 stage, the proportion of dry matter allocated to kernels under V12-HT, VT-HT, R2-HT, and R3-HT decreased by 26.48%, 27.68%, 13.67%, and 8.10%, respectively. In contrast, the proportions allocated to leaves increased by 24.64%, 32.91%, 12.50%, and 8.94%, whereas those allocated to stems increased by 27.96%, 25.25%, 12.68%, and 13.29%, respectively. Overall, high-temperature stress shifted dry matter allocation from reproductive organs to vegetative organs, with the most pronounced changes observed under V12-HT and VT-HT.

## 4 Discussion

Previous studies have shown that high-temperature stress significantly decreases chlorophyll content, RuBPCase and PEPCase activities, and photosynthetic capacity in maize, thereby limiting dry matter accumulation and grain yield formation (Zhang et al., 2008). High-temperature stress reduces photosynthesis through both stomatal and non-stomatal limitations. Stomatal limitation is generally characterized by stomatal closure accompanied by a reduction in Ci, whereas an increase in Ci together with a decline in Pn usually indicates that non-stomatal limitation predominates (Kong et al., 2016; Sharkey et al., 1982). In the present study, the simultaneous decrease in Pn and Gs, together with a marked increase in Ci, suggests that the reduction in photosynthetic performance was primarily associated with non-stomatal limitation, although the possible contribution of mesophyll conductance cannot be excluded. Moreover, the concurrent decline in SPAD value indicates that chlorophyll degradation may have further reduced the photosynthetic capacity of maize leaves. Collectively, these findings suggest that high-temperature stress impaired photosynthetic performance through coordinated disruption of chlorophyll maintenance and the photosynthetic apparatus rather than through stomatal limitation alone.

Chlorophyll fluorescence parameters are important indicators of PSII function. Among these parameters, Fv/Fm is widely used to evaluate the maximum photochemical efficiency of PSII and is highly sensitive to high-temperature stress (Sharkey and Zhang, 2010). Under high-temperature stress, the PSII reaction center is damaged, electron transport is inhibited, and less excitation energy is utilized for photochemical reactions. Consequently, excess excitation energy accumulates and is dissipated through qN as a photoprotective mechanism (Yu et al., 2023). In the present study, Fv/Fm, ΦPSII, and qP decreased significantly, whereas qN increased under all high-temperature treatments, indicating that high-temperature stress impaired PSII function by restricting electron transport, reducing photochemical efficiency, and enhancing thermal dissipation of excess excitation energy (Maxwell and Johnson, 2000). Moreover, the reductions in chlorophyll fluorescence parameters were generally greater at the V12 and VT stages than at the R2 and R3 stages, suggesting that the photosynthetic apparatus was more susceptible to high-temperature stress during these earlier developmental stages. Similar stage-dependent responses have also been reported by Zhang et al. (2022), who found that high-temperature stress imposed at the V12 or VT stage caused greater reductions in photosynthesis, dry matter accumulation, and grain yield.

Sun et al. (2017) showed that high-temperature stress disrupted the antioxidant balance system in maize leaves, resulting in MDA accumulation, enhanced membrane lipid peroxidation, decreased osmotic adjustment substances, and accelerated leaf senescence. To cope with oxidative damage, plants activate the antioxidant enzyme system, including SOD, POD, and CAT, which scavenges excess ROS and maintains cell membrane integrity (Su et al., 2026). Xu et al. (2024) further reported that SOD and CAT activities in ear leaves of different maize hybrids and their parental inbred lines decreased under high-temperature stress, whereas MDA content increased, although the magnitude of these responses differed among genotypes. Consistent with these reports, high-temperature stress at different developmental stages significantly reduced SOD, POD, and CAT activities while markedly increasing MDA content in the present study. These results indicate that when high-temperature stress exceeds the self-regulation capacity of plants, antioxidant enzyme activity declines, leading to the accumulation of MDA, disruption of membrane integrity, and accelerated leaf senescence. Moreover, the decline in photosynthetic performance occurred simultaneously with the reduction in antioxidant enzyme activity and the accumulation of MDA, suggesting that high-temperature stress first impaired the photosynthetic apparatus and triggered excessive ROS production. The weakened antioxidant defense system was unable to eliminate excess ROS efficiently, thereby further aggravating oxidative damage to the photosynthetic apparatus and forming a positive feedback loop that accelerated leaf senescence (Hu et al., 2023; Li et al., 2024). The reduction in antioxidant enzyme activity may also be associated with heat-induced inhibition of enzyme synthesis, reduced protein stability, or suppression of antioxidant enzyme gene expression, although these molecular regulatory mechanisms were beyond the scope of the present study and warrant further investigation (Hasanuzzaman et al., 2020). Collectively, these findings indicate that oxidative damage and photosynthetic impairment were closely coupled under high-temperature stress, jointly accelerating leaf senescence and limiting carbon assimilation.

**Figure 11:**
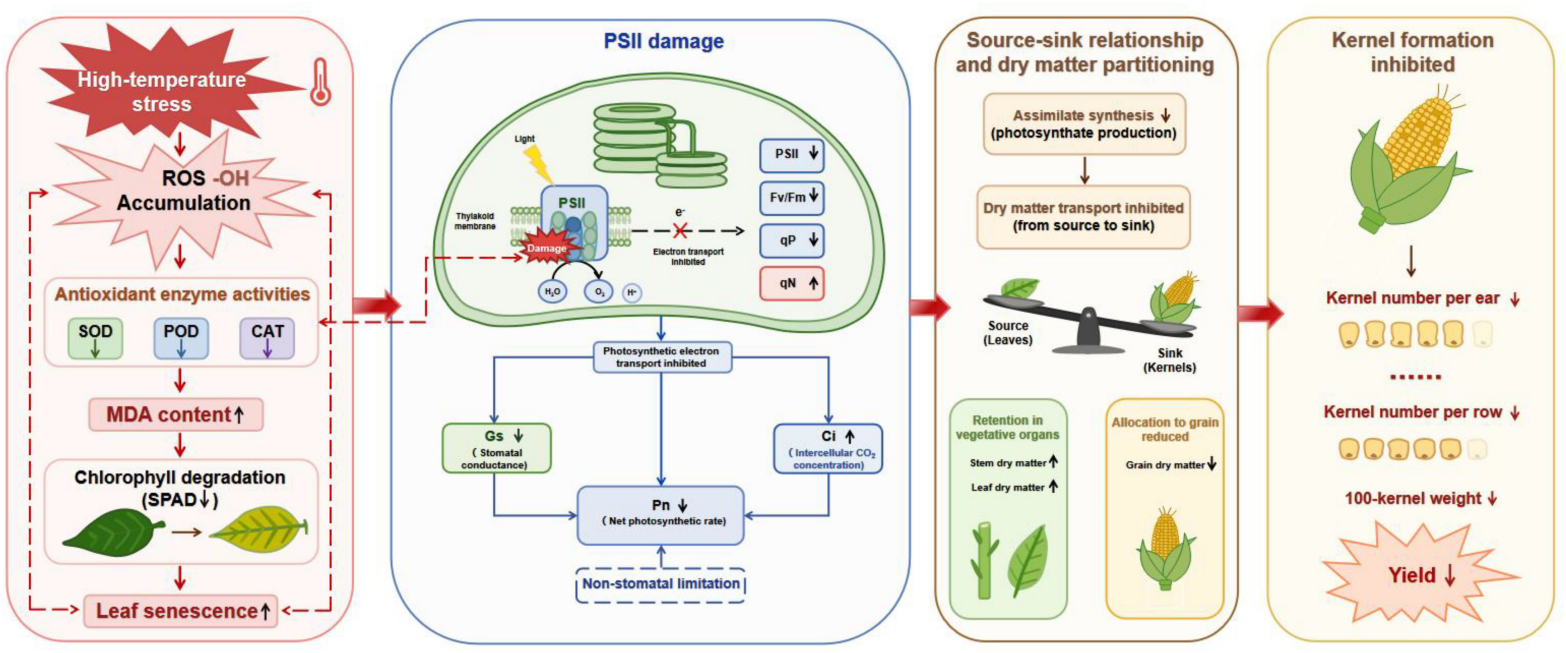
Proposed mechanism of high-temperature stress-induced leaf senescence and yield reduction in summer maize. Solid arrows indicate the proposed causal relationships based on the present results and previous studies, whereas dashed arrows indicate proposed positive feedback pathways.

Efficient grain filling requires not only strong photosynthetic capacity of functional leaves but also appropriate partitioning and efficient transport of assimilates from source to sink organs (Luo et al., 2009). Previous studies have shown that high-temperature stress significantly affects the growth and development of maize by reducing photosynthetic assimilate production, accelerating vegetative organ senescence, and increasing respiratory consumption, thereby decreasing the amount of assimilates available for grain filling and ultimately reducing grain yield (Tao et al., 2016; Edreira and Otegui, 2013). Yu et al. (2016) further reported that high-temperature stress altered dry matter distribution among maize organs, indicating that assimilate partitioning is highly sensitive to heat stress. Consistent with these findings, high-temperature stress significantly reduced dry matter accumulation and altered dry matter partitioning in the present study. The proportion of dry matter allocated to sink organs, including the cob and kernels, decreased, whereas that allocated to vegetative organs, such as stems, leaves, and husk leaves, increased. In addition, kernel number per row, kernel number per ear, 100-kernel weight, and grain yield all decreased significantly under high-temperature stress. These results suggest that high-temperature stress weakened source strength and reduced assimilate transport to developing kernels, thereby limiting kernel set and grain filling and ultimately reducing grain yield (**Figure 11**). In addition to reduced assimilate production, previous studies have suggested that changes in hormone signaling, particularly abscisic acid (ABA) and ethylene, also participate in regulating source–sink coordination under high-temperature stress, although these hormonal responses were not examined in the present study (Ma et al., 2023; Rosado-Souza et al., 2023). Similar developmental stage-dependent responses have also been reported in rice and wheat, indicating that disruption of source–sink coordination is a common physiological mechanism underlying heat-induced yield loss in cereal crops (Mohan et al., 2023).

## 5 Conclusions

In summary, high-temperature stress disrupted photosynthesis and antioxidant defense, aggravated oxidative damage, accelerated leaf senescence, and restricted assimilate transport to developing kernels, thereby reducing kernel number per ear, 100-kernel weight, and ultimately grain yield. The V12 and VT stages were more sensitive than the R2 and R3 stages. Accordingly, these stages represent key windows for mitigating high-temperature damage in maize. Targeted management strategies, such as canopy regulation or optimized plant density at V12 and sprinkler irrigation during VT to enhance pollination under heat stress, may help alleviate yield losses. Further studies integrating physiological and molecular analyses across a wider range of developmental stages are still needed to elucidate the regulatory mechanisms underlying stage-dependent responses of maize to high temperature.

## Data Availability

The original contributions presented in the study are included in the article/supplementary material, further inquiries can be directed to the corresponding author/s.
